# MinION Sequencing of Fungi in Sub-Saharan African Air and a Novel LAMP Assay for Rapid Detection of the Tropical Phytopathogenic Genus *Lasiodiplodia*

**DOI:** 10.3390/pathogens13040330

**Published:** 2024-04-17

**Authors:** Kevin M. King, Gail G. M. Canning, Jonathan S. West

**Affiliations:** Rothamsted Research, Harpenden AL5 2JQ, UK

**Keywords:** air sampling, community structure, diversity, drone, fungi, LAMP

## Abstract

To date, there have been no DNA-based metabarcoding studies into airborne fungi in tropical Sub-Saharan Africa. In this initial study, 10 air samples were collected onto Vaseline-coated acrylic rods mounted on drones flown at heights of 15–50 meters above ground for 10–15 min at three sites in Ghana. Purified DNA was extracted from air samples, the internal transcribed spacer (ITS) region was amplified using fungal-specific primers, and MinION third-generation amplicon sequencing was undertaken with downstream bioinformatics analyses utilizing GAIA cloud-based software (at genus taxonomic level). Principal coordinate analyses based on Bray–Curtis beta diversity dissimilarity values found no clear evidence for the structuring of fungal air communities, nor were there significant differences in alpha diversity, based on geographic location (east vs. central Ghana), underlying vegetation type (cocoa vs. non-cocoa), or height above ground level (15–23 m vs. 25–50 m), and despite the short flight times (10–15 min), ~90 operational taxonomic units (OTUs) were identified in each sample. In Ghanaian air, fungal assemblages were skewed at the phylum taxonomic level towards the ascomycetes (53.7%) as opposed to basidiomycetes (24.6%); at the class level, the Dothideomycetes were predominant (29.8%) followed by the Agaricomycetes (21.8%). The most common fungal genus in Ghanaian air was cosmopolitan and globally ubiquitous *Cladosporium* (9.9% of reads). Interestingly, many fungal genera containing economically important phytopathogens of tropical crops were also identified in Ghanaian air, including *Corynespora*, *Fusarium*, and *Lasiodiplodia.* Consequently, a novel loop-mediated isothermal amplification (LAMP) assay, based on *translation elongation factor*-1α sequences, was developed and tested for rapid, sensitive, and specific detection of the fungal phytopathogenic genus *Lasiodiplodia*. Potential applications for improved tropical disease management are considered.

## 1. Introduction

Most devastating staple calorie crop and economically valuable commodity crop diseases are caused by fungi and oomycetes; these pathogens are now emerging at an ever increasing pace due to a range of factors, including increased market globalization and climate change [1]. Given the clear threat posed by some phytopathogenic fungi to global food security, aerobiological research, particularly relating to fungi dispersed in air, can have many potentially important uses. These are not just restricted to monitoring for the early detection of economically important phytopathogens [2,3,4,5,6,7,8], or screening for fungicide resistance [9,10] but also include screening for human allergenic or pathogenic fungi [11,12] or for biosecurity and biodiversity monitoring [13,14,15]. Such research could clearly be of strategic importance to Sub-Saharan Africa, given that it is the least food-secure region in the world, and particularly in the context of the projected negative impacts from climate change and increased population growth expected there [16].

In the modern genomics era, genomic DNA-based approaches involving the study of genetic material from environmental samples are now routinely applied to investigate fungal community composition and diversity in diverse geographic/climatic parts of the globe ranging from the polar Antarctic [17] through to Mediterranean dust [18]. Such approaches have been utilized to gain novel insights into microbial (e.g., bacterial, fungal, oomycete, and viral) communities from a broad range of environmental sample types, including water, plants, soil, and air collected from different ecological regions of the world [19]. One such genomics-based approach now starting to be widely deployed is Oxford Nanopore’s MinION platform. This third-generation sequencing approach has several advantages over other sequencing technologies, including its portability, user friendliness, simplicity, rapid sequencing speed, and long read length; however, so far, the major limitation is its low read accuracy, although this is continually improving [20]. Indeed, the most recent Oxford Nanopore MinION flow cells (R10.4.1), when used with ligation sequencing kit V14 and using super accuracy basecaller settings, have a reported read accuracy of 99.5% when tested against sample Human HG002 DNA (https://nanoporetech.com/accuracy; accessed online, 13 March 2024).

Most aerobiological research, particularly involving genomics-based technologies, has been undertaken in developed countries including in Europe and North America e.g., [14]. By contrast, only extremely limited fungal aerobiological research has been undertaken to date in Africa, with most past work based on conventional culture-based approaches and the subsequent morphological identification of resultant cultures/spores; they have also predominantly focused on dusty or dessert air, for instance, in countries like Egypt, Mali, Nigeria, and Sudan [21]. For example, in dust samples collected from Mali (West Africa), based on conventional agar plate culturing of collected air, only three viable fungal genera were identified, predominantly *Cladosporium* (one of the most common and widespread fungi in air internationally [22] but also *Alternaria* and *Aspergillus* [21]. Ismail et al. [23] found that in air samples from arid/semi-arid desert sites in Egypt, 44 viable fungal genera were identified. Furthermore, such studies have focused on indoor environments such as schools and hospitals [24]. There are several limitations to such cultured-based studies including that not all fungal species are culturable on artificial agar media (or may have specific growth requirements); common and fast growing fungi (e.g., *Aspergillus*, *Cladosporium*) are likely to outgrow slower growing fungi and thus bias results; morphological identification of fungal cultures/spores is subjective and reliant on mycological expertise; and working with individual isolates (for which species-level identity is typically confirmed by sequencing of specific barcoding genes) means far less overall data are obtained than when applying sequence-based approaches to investigate fungal community structure in environmental samples.

The objectives of this study are (1) to use MinION metabarcoding sequencing to investigate in a first small-scale culture-independent study the airborne fungi present in tropical Sub-Saharan African air; and (2) to develop a first loop-mediated isothermal amplification (LAMP) assay for the rapid detection of *Lasiodiplodia*, an economically important genus that contains many tropical phytopathogens.

## 2. Methods

### 2.1. Air Sample Collection and DNA Extraction

**Figure 1:**
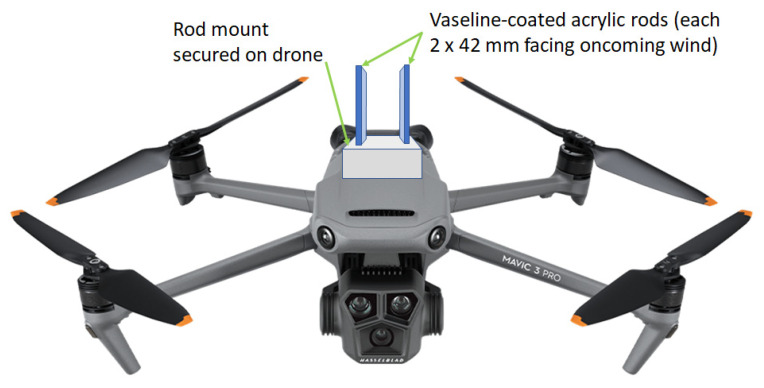
Schematic representation of Ghana air sampling onto Vaseline-coated acrylic rods mounted onto a Mavic air 3 drone (DJI, China).

Full details of the 10 tropical Ghanaian air samples, collected from three geographic locations, analyzed in this study are given in Table 1. For each drone flight, two Vaseline-coated acrylic rods were mounted (coated surface of 2 mm × 42 mm) facing oncoming air onto Mavic Air 3 drones (DJI, China) (Figure 1). During the 10–15-min flight, air particulates were deposited onto rods by impaction (corresponding to 0.3 to 0.56 cubic meters of air sampled, depending on flight speed and duration), after which they were placed into sterile 2 mL screwcap tubes and sent to Rothamsted Research (Harpenden, UK) for processing. Samples were stored at −20 °C prior to genomic DNA extraction and purification (both into 100 μL 1 × Tris-EDTA (TE) buffer), the latter using the EchoClean DNA Cleanup kit (Thistle Scientific, Rugby, UK); protocols were used as described in Vicentini et al. (2023) [10].

### 2.2. PCR Amplification of the ITS Region from Air Samples

The primers used in this study for metabarcoding amplicon sequencing had previously been successfully applied to a diverse range of ascomycete, basidiomycete, and mucoromycete fungi. These primers targeted the internal transcribed spacer (ITS) region, a marker widely used for the metagenomic profiling of fungal communities (despite inherent limitations such as variable gene copy number, differences in length that may result in PCR amplification bias, etc. [25]). The forward primer ITS1f-Kyo2 [26] binding towards the end of the Small SubUnit (SSU) 18S rRNA gene and the reverse primer LR3-I [27] binding within the Large SubUnit (LSU) 28S rRNA gene resulted in an amplified region primarily encompassing the ITS1, 5.8S, ITS2, and a partial region of LSU. PCR was carried out using PfuUltra II Fusion HS DNA Polymerase enzyme (Agilent Technologies, Stockport, UK), with each 20 μL reaction containing 2 μL reaction buffer (10× stock), 0.4 μL dNTPs (25 mM stock), 16.54 μL PCR-grade water, 0.08 μL each of primers ITS1f-Kyo2 (5′ TAGAGGAAGTAAAAGTCGTAA 3′) and LR3-I (5′ TGGTCCGTGTTTCAAGAC 3′) (100 μM primer stocks), 0.4 μL enzyme, and 0.5 μL purified genomic DNA. PCR conditions were an initial denaturation at 95 °C for 2 min; 35 cycles of denaturation at 95 °C for 20 s, annealing at 53 °C for 20 s, and extension at 72 °C for 1 min; followed by a final extension at 72 °C for 5 min; with a final hold at 4 °C. Successful PCR product amplification was confirmed by agarose gel electrophoresis.

### 2.3. MinION Amplicon Sequencing of Air Samples

Metabarcoding sequencing via MinION, including end-prep and PCR adaptor ligation and amplification steps, of PCR amplicons was carried out using a Native Barcoding kit 96 v14 (SQK NBD114.96 Oxford Nanopore Technologies, Oxford, UK) according to the manufacturer’s instructions. All individual samples were tagged with a unique barcode primer from the kit. Throughout, all DNA was quantified via a Qubit spectrometer (v.4.0; Invitrogen, Singapore) using a high-sensitivity (0–10 ng) kit. The total starting template DNA for each PCR amplicon sample used with the kit was 50–100 ng.

### 2.4. Downstream Bioinformatics Analyses

All sequencing runs used the minKNOW (v. 20.06.4) software package. Prepared libraries (11 μL) were loaded onto R10.0 (Spot-UN) MinION flow cells (Oxford Nanopore, Oxford, UK). Sequencing data were basecalled using the Metrichor 2D base-calling RNN for the SQK NBD114.96 workflow. The flow cell was run for 24 h, with super-high-accuracy basecalling enabled. Sequence reads were assigned to bins for each individual sample based on barcode primer tag identity; the N50 of obtained reads was ~1.2 Kb. The program was set to trim barcodes at both ends. FASTQ reads were classified as either ‘pass’ or ‘fail’ with a Q-value set at 20 (i.e., a 1% error rate), with minimum and maximum read lengths set at 0.4 Kb and 2.0 Kb, respectively. For each of the ten samples, 4000 FASTQ reads were obtained; sequence data were deposited in the NCBI under BioProject PRJNA1095819 (biosamples SAMN40733751–SAMN40733760). The sequences were imported into GAIA metagenomics cloud-based software (v2.02, Sequentia Biotech, Barcelona, Spain) [28,29] for subsequent bioinformatics analyses.

In GAIA, sequence reads underwent further quality checking/trimming and adaptor removal using default settings (only 1–3 reads removed per sample, i.e., 3997–4000 reads remaining), with high-quality reads subsequently mapped against the ‘Amplicon ITS1 and ITS2 (released 2020)’ custom-made reference database. Reads were classified to the most specific taxonomic level possible (species, genus, family, order, class, phylum, and domain) using an in-house Lowest Common Ancestor (LCA) algorithm (a minimum threshold of 70% identity and coverage was used by GAIA for read classification). In the present study, sequence datasets were analyzed at the genus taxonomic level; if this was not achieved in GAIA, the most specific taxonomic level obtainable was assigned to a particular OTU (e.g., domain, phylum, class, order, and family). Within GAIA, rarefaction plots were obtained using the vegan software package; alpha and beta diversities were calculated using phyloseq, which also generated downstream images and plots (e.g., principal coordinate analyses; PCoAs). Pairwise comparisons of pooled beta diversity Bray–Curtis dissimilarity values were carried out using ANOVA. Alpha diversity indices for samples, including numbers of OTUs, plus Chao1, Fisher, Shannon, and Simpson indices, were analyzed using Student’s *t* tests. Finally, datasets were examined using differential abundance analysis via DESeq2, with false discovery rates (*FDR*) considered statistically significant at *p* < 0.05.

### 2.5. Design, Validation, and Application of Lasiodiplodia LAMP Assay

Design: A LAMP assay primer set was designed for the detection of *Lasiodiplodia* spp. based on *translation elongation factor*-1α (*TEF-1α*) DNA sequences. A primer set was designed based on in silico analyses to target multiple species within the genus *Lasiodiplodia*: *L. parva* (GQ469904, MK495375); *L. pseudotheobromae* (OM934811, MN638768, MT408906, OM934806, OM934811, KX528572); and *L. theobromae* (MW591893, EU012398, GQ469897, MT086514). The primers were designed based on sequence differences so as not to target closely related fungi including *Botryosphaeria dothidea* (KF778974, KF778975, AY236898); *Diplodia mutila* (KF778979, KF778981, MW591887, AY573219), *D. pseudoseriata* (EU863181), and *D. seriata* (MH908100, AY573220, KF778986, KF778987); and *Neofusicoccum arbuti* (KF531792, MW591899), *N. mediterraneum* (GU251308, KF779001, KF779003), and *N. parvum* (KF779044, KF779045). The primer sequences designed were LasEF1A F3: 5′ GCCTTATCGCTCTGGTGAG 3′; LASEF1A B3: 5′ TGTAGTGGGGCGCGTTAG 3′; LASEF1A FIP: 5′ AAGTGCGGTCATTTTGCCGAACTTTTTCGTGGTGGGGTTTGG 3′; LASEF1A BIP: 5′ GGTTTTTTTGCGACCGGCGTCGTACGACGGTACATGAGTG 3′; and LASEF1A LOOPB: 5′ CTCCCCACTAGCGAAAAATGCTCTG 3′.

LAMP assay reaction conditions: Each 15 μL reaction contained 0.3 μL FIP (100 μm primer stock), 0.3 μL BIP (100 μm), 0.15 μL LoopF (100 μm), 0.15 μL LoopB (100 μm), 0.3 μL F3 (10 μm), 0.3 μL B3 (10 μm), 7.5 μL isothermal mastermix (Optigene, 2× concentrate), 1 μL template genomic DNA (10 ng), and 5 μL PCR-grade water. Reaction conditions were a constant 65 °C for 30 min (FAM fluorescence being measured every 30 s); followed by a dissociation curve step at 95 °C for 1 min; 55 °C for 30 s; and 95 °C for 30 s. Two technical replicates were run for each sample tested using the Mx3000P v. 4.9 qPCR system (Agilent, Stockport, UK (previously Stratagene); a no-template water control was included for each run. Data were analyzed on a log scale with the cycle threshold (*C*_t_) adjusted manually to the exponential phase of amplification curves.

Evaluation of the *Lasiodiplodia* LAMP assay: The new LAMP assay primer set was initially tested for specificity against DNA from a panel of fungal isolates (Table 2), including representative key economically important species from the genus *Lasiodiplodia* (*L. pseudotheobromae*/*L. theobromae*) and other related fungi causing diseases to various tropical or temperate crops. The assay was further tested for sensitivity by screening in duplicate against DNA (quantified by *Qubit* 4.0 Fluorometer) of *L. pseudotheobromae* isolate CBS116459, with varying amounts of genomic DNA added per reaction as follows: 1 ng, 100 pg, 50 pg, 25 pg, 12.5 pg, 6.25 pg, 5 pg, 2 pg, and 1 pg. Samples were considered to test positive for *Lasiodiplodia* if amplification was detected prior to 60 min with clear single dissociation peaks.

Application of *Lasiodiplodia* LAMP assay to air samples: Purified genomic DNA (2 μL included in each reaction) isolated from the ten Ghanian air samples used previously in MinION metabarcoding sequencing (21GH06, 08, 10, 12, 14, 15, 16, 17, 18, 20; Table 3) was screened in quadruplicate against the new LAMP assay. Five of these ten samples had tested positive for *Lasiodiplodia* DNA based on MinION sequence data (Table 3). Samples were considered to test positive for *Lasiodiplodia* if all four technical replicates amplified prior to 2 h with single clear dissociation peaks evident.

## 3. Results

### 3.1. Principal Coordinate Analyses (PCoAs) and Beta Diversity of Ghanian Air Samples

**Figure 2:**
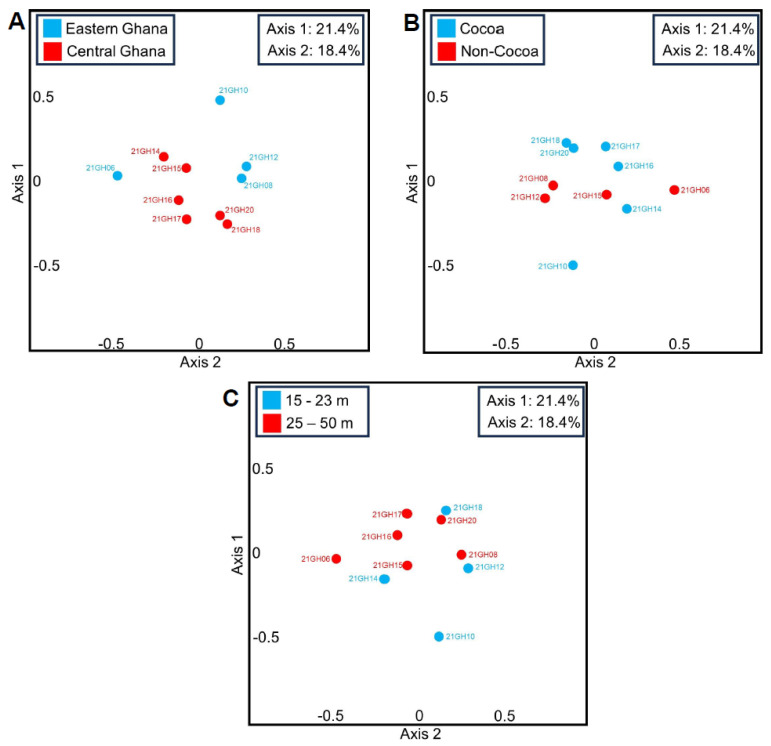
Principal coordinate analysis (PCoA) plots based on Bray–Curtis dissimilarity values (beta diversity) for fungal OTUs. These were assigned (at genus taxonomic level) with GAIA software using MinION third-generation amplicon sequencing (ITS) reads obtained using air samples collected in Ghana (Aburi, Assin Fosu, and Koforidua regions). Data were analyzed based on (**A**) geographic location (east vs. central Ghana), (**B**) crop above which air was sampled (cocoa vs. non-cocoa), and (**C**) height above ground (15–23 m vs. 25–50 m).

In the present study, PCoA based on Bray–Curtis dissimilarity values revealed no distinct clustering of fungal OTUs in ten Ghanian air samples based on geographic location (east vs. central Ghana; Figure 2A), vegetation type (cocoa vs. non-cocoa; Figure 2B), and sampling height above ground (15–23 m vs. 25–50 m; Figure 2C). Pairwise comparisons using ANOVA of pooled Bray–Curtis dissimilarity values of Ghana air samples found no significant differences based on location (i.e., east vs. east, east vs. central, and central vs. central) (*F*(2,42) = 1.06, *p* = 0.35), vegetation type (i.e., cocoa vs. cocoa, cocoa vs. non-cocoa, non-cocoa vs. non-cocoa) (*F*(2,42) = 0.01, *p* = 0.99), or sampling height above ground (i.e., 15–23 m vs. 15–23 m, 15–23 m vs. 25–50 m, 25–50 m vs. 25–50 m) (*F*(2,42) = 1.53, *p* = 0.23).

### 3.2. Alpha Diversity in Ghanian Air Samples

Full alpha diversity indices values (Chao1, Shannon, Simpson, and Fisher), and results of statistical T-tests, are presented in Table 4. The inspection of rarefaction plots produced in GAIA suggested that the number of OTUs identified as a function of the number of reads had either plateaued or begun to plateau, suggesting that the majority of the species richness had been captured (Figure S1). A mean value of 90.1 OTUs (standard error of mean = 14.7) was identified in each of the ten Ghanian air samples tested. There were no statistically significant differences (*p* > 0.05) in alpha diversity, in terms of either the number of OTUs detected or any of the four individual calculated indices, between air samples collected from different locations (east vs. central Ghana), the vegetation type above which the air was sampled (cocoa vs. non-cocoa), or the height above ground (15–23 m vs. 25–50 m).

### 3.3. Predominant OTUs in Ghanaian Air

**Figure 3:**
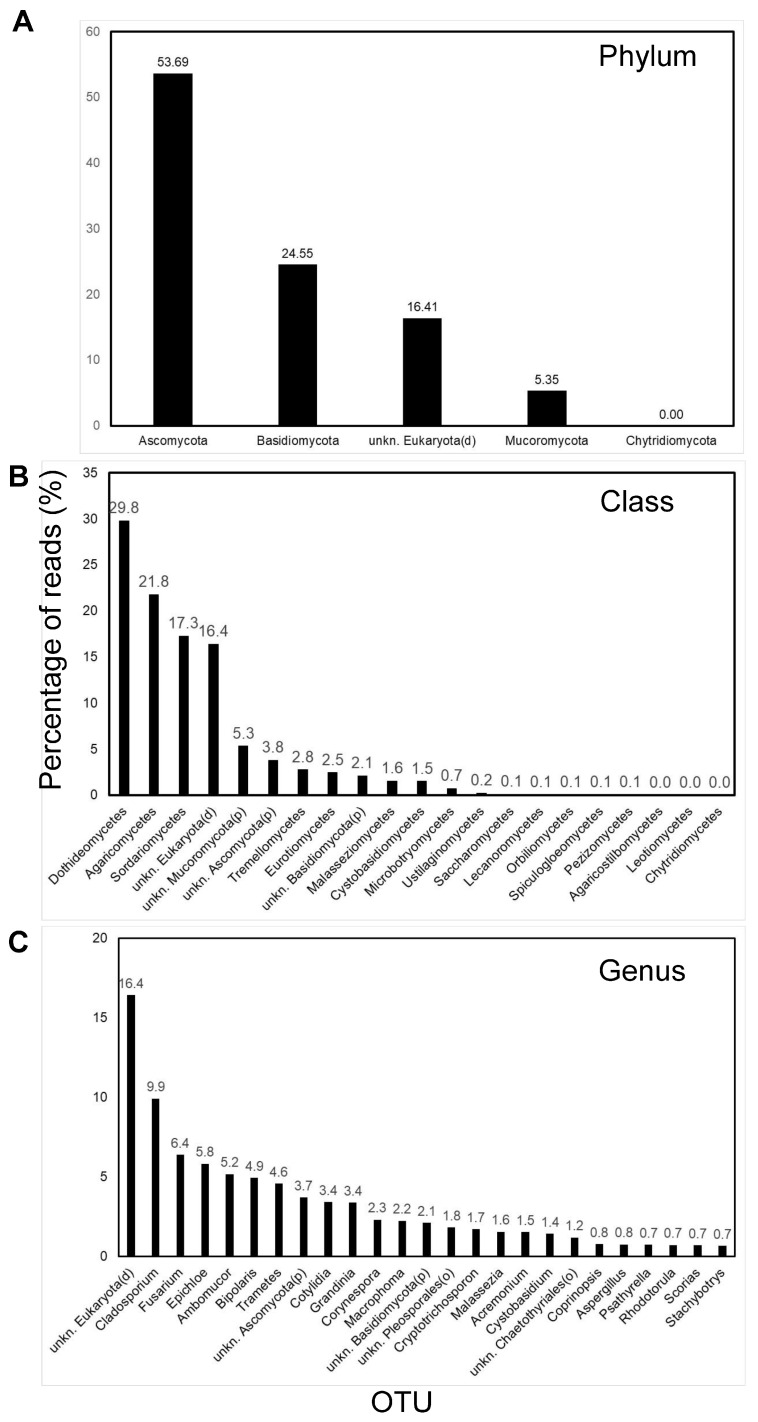
Fungal community structure for air samples from Ghana (*n* = 10) as determined via GAIA software of third-generation MinION amplicon (ITS) sequence reads. Shown are the percentages of reads from the UK and Ghana assigned to (**A**) phylum, (**B**) class, and (**C**) genus taxonomic levels.

In Ghanaian air samples, at the phylum taxonomic level, most of the fungal OTUs identified using GAIA were ascomycetes (53.7 of reads), followed by basidiomycetes (24.6%) and mucoromycetes (5.4%) (Figure 3A). A minority (16.4%) of reads were identified as unknown eukaryotes (at the domain taxonomic level), and these were excluded from further consideration here. At the class taxonomic level, the Dothideomycetes were predominant (29.8%), followed by Agaricomycetes (21.8%) and Sordariomycetes (17.3%) (Figure 3B). In total, 335 OTUs were identified in the ten combined Ghanian air samples when analyzed at the genus taxonomic level, with the top five OTUs contributing approximately a third of all obtained reads comprising *Cladosporium* (9.9% reads), *Fusarium* (6.4%), *Epichloe* (5.8%), *Ambumucor* (5.2%), and *Bipolaris* (4.9%) (Figure 3C). When inspected at the species taxonomic level, eight different OTUs were identified for *Cladosporium*, with the most common being, by far, *C. cladosporioides* (9.4%).

At the genus taxonomic level, a number of the top ten OTUs identified were potentially important phytopathogens including *Cladosporium*, *Fusarium*, *Epichloe*, *Bipolaris*, and *Corynespora* (2.3% of reads). For the genus *Corynespora* two species were indicated with the non-pathogenic *C. smithii* appearing more common (2.2%) than the pathogenic *C. cassiicola* (0.1%). For the genus *Bipolaris*, at the species level, four OTUs were identified, with the most common indicated being the phytopathogens *B. maydis* (3.3%) and *B. sorokiniana* (1.6%). For the *Fusarium* genus, 16 OTUs were indicated at species level, with the predominant being *F. equiseti* (4.2% of reads). Although identified less frequently in Ghanian air, a great many other fungal genera were also identified that included major economically important phytopathogens such as *Cercospora* (0.3%), *Lasiodiplodia* (0.3%), *Macrophoma* (2.2%), and *Pseudocercosporella* (0.5%). Also identified were several fungal genera that included pathogens of humans (e.g., *Aspergillus*, 0.8%) and/or animals (e.g., *Pithomyces*, 0.7%).

There were relatively few fungal genera that were statistically over-represented (*Psathyrella*, *Epicoccum*, *Malassezia*; *p* < 0.05) or under-represented (*Cystobasidium*, unknown basidiomycota (phylum level), Cystobasiomycetes (class level), *Curvularia*, *Surculiseries*, *Phyllozyma*, *Eupenidiella*; *p* < 0.05) in air sampled from eastern Ghana compared to central Ghana. Next, there were also only small numbers of fungal genera that were statistically over-represented (*Solicoccozyma*, *Pseudozyma*, *Rhodotorula*, *Pyrenochaeta*; *p* < 0.05) or under-represented (*Pyrenochaetopsis*, *Mycosphaerella*, *Pseudotaeniolina*, *Neofusicoccum*, *Thyronectria*; *p* < 0.05) in air sampled above non-cocoa fields compared to cocoa crops. Lastly, there were, once again, a small number of fungal genera that were statistically over-represented (*Solicoccozyma*, *Pyrenochaeta*, *Pseudozyma*; *p* < 0.05) or under-represented (*Exophiala*, *Phyllozyma*; *p* < 0.05) in air sampled at 15–23 m above ground versus air sampled at a slightly higher level above ground at 25–50 m.

### 3.4. Design, Validation, and Application of Lasiodiplodia LAMP Assay

The developed assay was specific to genomic DNA of the *Lasiodiplodia* spp. tested (*L. theobromae* and *L. pseudotheobromae*), with a clear amplification signal and corresponding dissociation curve produced after ≈14–16 min (Table 2). No amplification signal was evident prior to 80 min for a panel of DNA from a broad range of other closely related plus more distantly related fungi, with the exception of three *Diplodia* spp. isolates that were amplified between 50 and 60 min. A single dissociation peak of 91.7 °C was evident for all positive samples, indicating the specific amplification of target DNA. The developed assay could successfully detect down to at least 6.25 pg of *L. pseudotheobromae* DNA (all within 60 min). When applied to ten Ghanian air DNA samples, of the five samples for which *Lasiodiplodia* had been identified as present based on MinION sequencing, a single sample tested positive using LAMP (Table 3). This sample (21GH12) contained the greatest proportion of *Lasiodiplodia* DNA (1.31% of reads), and all four technical replicates tested positive using the new LAMP diagnostic for this sample.

## 4. Discussion

In tropical Sub-Saharan Ghanian air samples collected in the present study, based on metabarcoding analyses of fungal ITS1 and ITS2 rDNA sequences, ascomycetes were more abundant (53.7% of reads) than basidiomycetes (24.6% of reads), followed by the mucoromycetes (10.3%). At the class taxonomic level, Dothideomycetes were predominant in tropical Ghanaian air (29.8% of reads) followed by Agaricomycetes (21.8% of reads) and Sordariomycetes (17.3% of reads). It should be noted that around 16.4% of reads in tropical Ghanian air were identified as unknown Eukaryotes, a result likely attributable, at least in part, to less fungal sequence data being available for the former in online sequence repositories; only limited mycological research has been undertaken in Africa, most of which is based on traditional culture-based (morphological identification), as opposed to molecular (sequence-based), identification [21]. These initial sequencing results, admittedly based on a relatively small sample size, therefore also highlight the importance of future molecular mycological research in Africa to fill these knowledge gaps.

Based on PCoA plots and statistical analyses of beta diversity (Bray–Curtis dissimilarity) values, no evidence for fungal community structuring was evident based on either geographic location (east vs. central Ghana, sites separated by ~120 km), vegetation type (cocoa vs. non-cocoa), or height above ground (15–23 m vs. 25–50 m). Thus, fungal community composition was largely homogenous across all ten Ghanian air samples tested. This would be expected due to the sample heights in this study of 15–50 m above ground, representing air that would have been well mixed by turbulence. It is known that air sampled closer to the ground can be expected to bias sampling towards more local inoculum sources (i.e., less diverse), whereas air sampled at a higher elevation can be expected to include more mixed air containing particulate matter released over a larger geographic area (i.e., more diverse) [14,30,31,32].

A key finding of this study is therefore that fungal communities in the three Ghanaian sites examined were largely homogenous, despite them being separated by distances up to 120 km apart. Indeed, only very few OTUs, when analyzed at the genus taxonomic level, were over/under-represented in any of the different conditions, indicating some effect of more local sources of these species. Similar results were reported by Nicolaisen et al. [14] who found no clear evidence for the structuring of fungal communities in air samples collected ~10 m above grove at three sites in northwestern Europe that had similar land-use and climatic conditions. It is probable that sampling fungal diversity at a range of tens to hundreds of kilometers can be achieved quickly by sampling air at higher elevations (e.g., 15–50 m) above ground. However, to obtain more specific information on fungal community structure at a local level (for instance a single crop field), the air to be sampled should be collected closer to ground level and for longer time periods (to ensure full diversity is captured given that this lower-level air is likely to be less mixed and diverse [14]). The latter sampling strategies are likely to inform us on risks posed by fungal pathogens to animal/plant health at a more local level, enabling more targeted disease management strategies.

An inspection of rarefaction curves clearly indicated OTU numbers as a function of read number, which were either beginning or had already begun to plateau in the Ghanaian air samples, suggesting that the sampling duration and number of reads used in this study were sufficient. One objective of this study was to undertake preliminary investigations into the fungal aerobiome of tropical Sub-Saharan Africa, but future work should incorporate more intensive air sampling to ensure greater species diversity is captured. This is particularly the case for the Ghanaian air samples collected by drone-mounted samplers for which flight times were only 10–15 min in length; longer flight times at faster speeds would increase the amount of air particulate sampled and hence the species richness captured. Nevertheless, given that rarefaction curves were approaching plateau, it appears that the sampling of air in tropical environments at elevated altitudes (15–50 m) via drone-mounted cyclone samplers, even for relatively short periods of time (10–15 min), is sufficient to capture much of the fungal alpha diversity present. Indeed, of the 10 such Ghanaian air samples, a mean of ~90 OTUs were identified in each sample (analyzed at the genus taxonomic level), showing the considerable fungal diversity present in tropical air. There were no significant statistical differences in the number of OTUs, and for the four different alpha diversity indices used between the ten air samples collected in terms of geographic location, vegetation type, or height of sampling above ground.

In the present research, the most common fungal genus identified in Ghanaian air was *Cladosporium* (9.9% of reads). These results are consistent with numerous other studies that have confirmed the widespread ubiquitous distribution of this fungal genus throughout many geographic regions of the world [22]. *Cladosporium* has a cosmopolitan distribution and represents the most common fungi present in air, also having the potential to act as important animal and plant pathogen [33]. Airborne spores of *Cladosporium* have previously been studied in other African countries including Nigeria and South Africa, due to their economic and medical importance as pathogens in this geographic region of humans and crops [34,35].

In Ghanaian air, many economically important genera of phytopathogenic fungi were identified. One notable such genus was *Corynespora*, with both the saprotrophic *C. smithii* and pathogenic *C. cassiicola* indicated in this study using GAIA at the species taxonomic level [36]. The latter species is an economically important plant pathogen worldwide with a broad plant host range [37]. For instance, rubber represents a key tree crop grown commercially throughout Ghana but Corynespora Leaf Fall (CLF), caused by *C. cassiicola*, has negatively impacted many rubber-growing regions of Africa and Asia [38]. In addition, *Fusarium* was the second most common fungal genus in Ghanaian air (mean 6.4% of sequence reads), with the predominant indicated species being the phytopathogenic *F. equiseti* (4.2%), which is known to occur on maize, which is a crop grown in clearings within the study region. *Fusarium* species are known to cause diseases on many plant hosts throughout the world and produce mycotoxins that can impact animal and human health. Also of note was the sporadic detection of the genus *Lasiodiplodia* (present in 5 out of 10 samples based on MinION sequencing) in Ghanaian air, albeit at relatively low levels (ranging from 0.12% up to 1.31% of reads). *Lasiodiplodia* is a notable fungal genus as it includes many economically important phytopathogens, particularly of tropical crops.

Cocoa is an economically important crop in West Africa, a region that produces a substantial proportion (~3 M tons) of the world (~4.4 M tons) crop, primarily by smallholder growers along the Ivory Coast (~1.7 M tons) and in Ghana (0.9 M tons) [39]. Conventional fungal isolation studies by Adu-Acheampong & Archer [40] identified both *Fusarium* spp. and *Lasiodiplodia* spp. (mostly *L. pseudotheobromae*) as the fungal genera most often associated with diseased cocoa stems in Ghana, with broadly similar results from a study in Nigeria [41]. Numerous *Fusarium* spp. have been reported to cause diseases of cocoa, but also to other economically important food and commercial crops in Ghana and elsewhere in west Africa. These include the root rot disease of cassava (*Manihot esculenta*) [42,43], and a range of cereal grain crops such as *Zea mays* (maize), *Oryza sativa* (rice), and *Sorghum* spp. (sorghum) [44]. In the present study, the most indicated *Fusarium* species identified in Ghanaian air when examined in GAIA at species taxonomic level was *F. equiseti*, a reported phytopathogen of numerous plant hosts in many countries including Ghana e.g., [45]. *Lasiodiplodia* spp. have been reported as increasingly important cocoa pod pathogens in several African countries, such as Cameroon, and now represent a constraint to African production [46]. *Lasiodiplodia* spp. were also found to be the predominant fungi isolated from diseased cocoa stems in other parts of the world, including Indonesia and the Philippines [47].

Given the detection of *Lasiodiplodia* spp. DNA in five of the Ghanian air samples examined using MinION-based metabarcoding, the clear economic importance of this fungal genus to a broad range of tropical crops, and the fact that no such LAMP assay was available, efforts were made here to develop such an assay for the detection of this fungal genus. The new LAMP assay, based on *translation elongation factor-1α* (*TEF-1α*) sequences, enabled rapid plus specific detection (~14–16 min) of *Lasiodiplodia* genomic DNA; other fungal species, including related tropical crop pathogens (*Diplodia*, *Dothiorella*, *Neofusicoccum*) and more distantly related temperate crop pathogens (*Plenodomus*, *Sclerotinia*, *Zymoseptoria*), were not detected prior to at least 50–60 min (usually 80 min). The developed assay was sensitive, being able to detect down to at least 6.25 pg of *L. pseudotheobromae* DNA; it can be used for the rapid confirmation of fungal cultures as *Lasiodiplodia* spp. Moreover, subject to further testing, it could likely be applied to determine the presence/absence of the genus in heavily infected plant tissues (e.g., lesions on fruit) for improved disease management, potentially by stakeholders (e.g., growers) under field-conditions. We further show here that the new LAMP diagnostic could also be applied to air environmental samples, although of the five *Lasiodiplodia*-positive samples (based on MinION metabarcoding), only the single sample containing the largest proportion of *Lasidiploda* reads tested positive with LAMP. These discrepancies could potentially be explained by a low copy number of the target organism in some samples, potentially coupled with the non-homogeneity of the DNA samples used.

Limited aerobiological research has been undertaken in Africa to date [21], almost always using traditional culturing-based approaches combined with morphological approaches and performed in (or immediately around) indoor environments such as hospitals [24]. To the best of the authors’ knowledge, this study represents the first study to apply metabarcoding sequence-based approaches, in this case using MinION third-generation sequencing, to investigate airborne fungal community structure and diversity in Sub-Saharan Africa. Many fungi that cause economically important plant diseases were identified in the tropical air sampled in this study, and, in future, more local air sampling of individual crops/fields could aid in early pathogen detection and in enabling improved crop protection strategies, particularly for local smallholder growers in food-insecure regions such as Sub-Saharan Africa [16]. The utilization of such DNA-based approaches will likely underpin future fungal aerobiological research, including aiding in the development of new molecular diagnostics (such as the LAMP assay in the present study) for use in pathogen surveillance, particularly as sequencing technology becomes more portable, accurate, and affordable, and more comprehensive fungal sequence databases become available.

## Figures and Tables

**Table 1:** Details of air samples from Ghana (onto Vaseline-coated acrylic rods mounted onto DJI Mavic Air 3 drones) used in this study.

Sample Code	Sample Date ^a^	Origin (Ghana)	Region	Sampling Height above Ground (m)	Drone Flight Speed (Kph)	Air Sampling Duration (min)	Sampling Site Notes	FASTQ Reads Obtained
21GH06	5 June 2021	Aburi	East	25	40	10	Citrus plantation	4000
21GH08	5 June 2021	Aburi	East	25	30	10	Citrus plantation	4000
21GH10	12 June 2021	Koforidua	East	20	40	15	Cocoa farm	4000
21GH12	12 June 2021	Koforidua	East	23	30	15	General vegetation	4000
21GH14	16 June 2021	Assin Fosu	Central	20	20	15	Cocoa farm	4000
21GH15	16 June 2021	Assin Fosu	Central	35	40	15	Palm plantation	4000
21GH16	16 June 2021	Assin Fosu	Central	30	20	15	Replanted cocoa trees	4000
21GH17	16 June 2021	Assin Fosu	Central	25	30	15	Cocoa farm	4000
21GH18	10 August 2021	Assin Fosu	Central	15	30	15	Cocoa farm	4000
21GH20	10 August 2021	Assin Fosu	Central	50	~40	15	Extended cocoa farm	4000

^a^ All Ghana (GH) air samples were collected from different farms at around noon.

**Table 2:** Fungal isolates used for specificity testing of the new *Lasiodiplodia* spp. LAMP assay.

Fungal Species	Isolate Code	Host	Source	Result ^5^
*Diplodia mutila*	Bot-2017-DM21-apple	Apple	^1^	Negative
*Di. mutila*	Bot-11-walnut	Walnut	^1^	Negative
*Di. mutila*	Bot-15-walnut	Walnut	^1^	Negative
*Di. mutila*	Bot-16-hazelnut	Hazelnut	^1^	Negative
*Di. sapinea*	No data (FR)	No data	^2^	Negative
*Di. seriata*	Bot-2018-S3-apple	Apple	^1^	Negative
*Di. seriata*	Bot-09-grape	Grape	^1^	Negative
*Dothiorella sarmentorum*	Bot-12-walnut	Walnut	^1^	Negative
*Lasiodiplodia pseudotheobromae*	CBS116459	Gamhar tree (Costa Rica)	^3^	Positive (~16 min)
*Las. theobromae*	Bot-2017-LT6-apple	Apple	^1^	Positive (~14.5 min)
*Las. theobromae*	CBS164.96	Fruit (Papua New Guinea)	^3^	Positive (~16 min)
*Neofusicoccum arbuti*	Bot-2017-NA5-apple	Apple	^1^	Negative
*Neo. parvum*	Bot-10-grape	Grape	^1^	Negative
*Neo. parvum*	Bot-13-walnut	Walnut	^1^	Negative
*Neo. parvum*	Bot-14-blueberry	Blueberry	^1^	Negative
*Plenodomus lingam*	22SURREY03	Oilseed rape (UK)	^4^	Negative
*Sclerotinia sclerotiorum*	SS1	Rapeseed mustard (UK)	^4^	Negative
*Zymoseptoria tritci*	80.4	Wheat (UK)	^4^	Negative

^1^ DNA kindly provided by Gonzalo Diaz Ulloa (University of Talca, Chile). ^2^ Cultures kindly supplied by Ana Pérez-Sierra (Forest Research, UK). ^3^ Cultures obtained from the CBS culture collection (Westerdijk Fungal Biodiversity Institute, The Netherlands). ^4^ Isolate from the plant pathogen fungal collection at Rothamsted Research (UK). ^5^ Negative: no amplification signal prior to at least 80 min, except for three *Diplodia* isolates that were amplified between 50 and 60 min (underlined).

**Table 3:** Application of *Lasiodiplodia* LAMP assay to ten Ghanian air samples.

Air Sample ID	MinION Metabarcoding Result		LAMP Assay Result
	Proportion of Reads (*Ladiodiplodia* Genus)	Result	Overall Result
21GH06	0	Negative	Negative
21GH08	0.25%	Positive	Negative
21GH10	0.13%	Positive	Negative
21GH12	1.31%	Positive	Positive
21GH14	0	Negative	Negative
21GH15	0.22%	Positive	Negative
21GH16	0.75%	Positive	Negative
21GH17	0	Negative	Negative
21GH18	0	Negative	Negative
21GH20	0	Negative	Negative

**Table 4:** Alpha diversity of Ghanian air samples determined at the genus taxonomic level in GAIA.

	Mean Value (Standard Error of Mean)				
	OTUs	Chao1	Shannon	Simpson	Fisher
**Ghana combined data (N = 10)**	90.1 (14.7)	127.0 (20.2)	2.5 (0.2)	0.8 (0)	17.5 (3.7)
Location:					
Eastern Ghana (N = 4)	79 11.6)	102.1 (14.5)	2.4 (0.2)	0.8 (0)	14.1 (2.4)
Central Ghana (N = 6)	97.5 (23.9)	143.7 (31.7)	2.6 (0.2)	0.8 (0)	19.8 (6.0)
*t*-test (*p* value)	0.6	0.3	0.6	0.9	0.5
Crop below:					
Cocoa (N = 6)	76.8 (5.9)	111.1 (9.9)	2.4 (0.1)	0.8 (0)	14.2 (0.9)
Non-Cocoa (N = 4)	110 (36.4)	151 (50)	2.7 (0.4)	0.9 (0)	22.4 (9.4)
*t*-test (*p* value)	0.3	0.4	0.5	0.4	0.3
Height above ground:					
15–23 m (N = 4)	79.3 (9.4)	100.8 (10.6)	2.3 (0.2)	0.8 (0)	15.1 (1.4)
25–50 m (N = 6)	97.3 (24.3)	144.5 (32.0)	2.7 (0.24)	0.9 (0)	19.1 (6.3)
*t*-test (*p* value)	0.6	0.3	0.3	0.1	0.6

## Data Availability

Data are shown in the Supplementary Excel File or are available on request from the authors.

## Supplementary Materials

The following are available online at https://www.mdpi.com/article/10.3390/pathogens13040330/s1, File S1. List of OTUs (Operational Taxonomic Units) identified in each of the ten Ghana air samples (see Table 1) given at the phylum, class, order, family, genus, and species taxonomic levels. Data are presented as the percentage of total reads (~4000) and were obtained using the GAIA software package.
