# Supplementary figures and images for: MinION Sequencing of Fungi in Sub-Saharan African Air and a Novel LAMP Assay for Rapid Detection of the Tropical Phytopathogenic Genus Lasiodiplodia
Source: Pathogens. 2024 Apr 17;13(4):330. doi: 10.3390/pathogens13040330 (PMC11053906; doi:10.3390/pathogens13040330)

OTU number

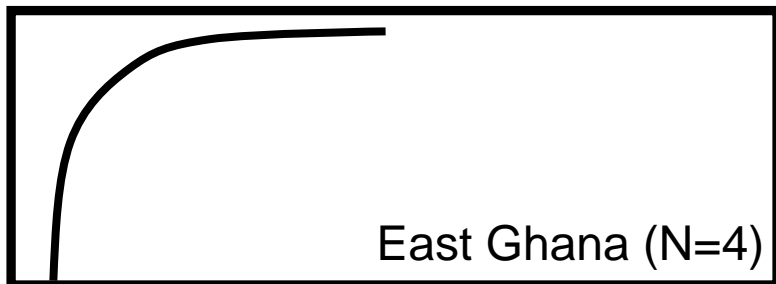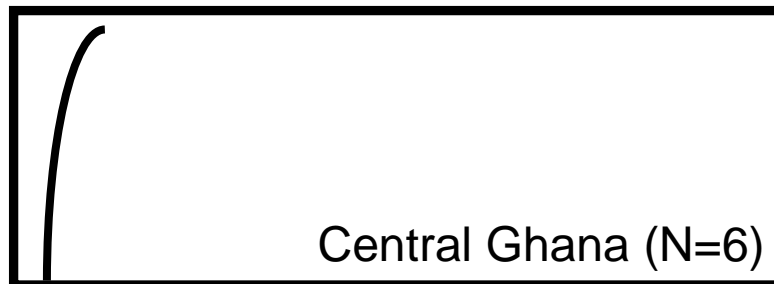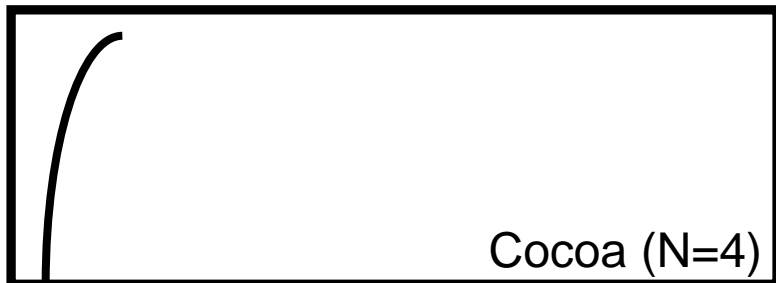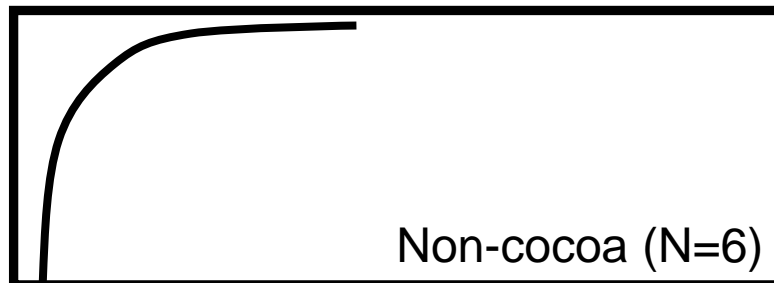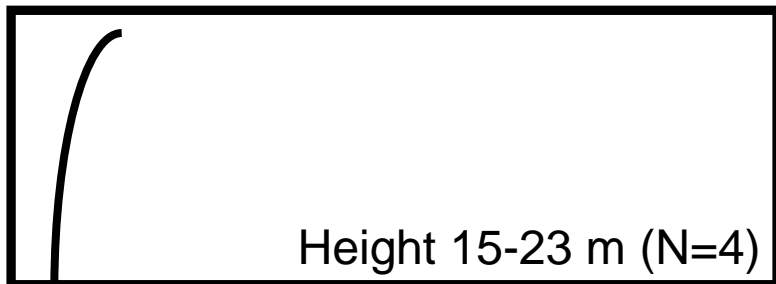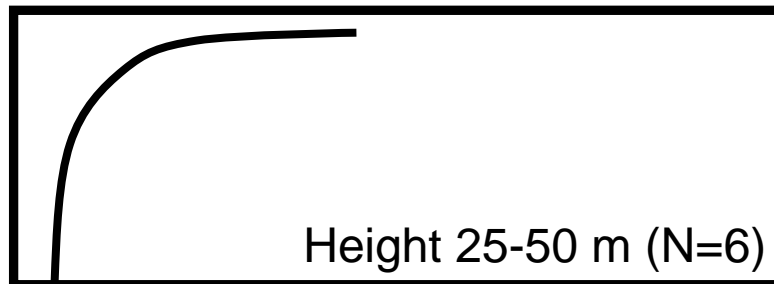

Read number

Supplement: Supplementary file 1 [file pathogens-13-00330-s001.zip › File S1/Figure S1.pdf]
